# Preclinical Data on the *Gardnerella*-Specific Endolysin PM-477 Indicate Its Potential to Improve the Treatment of Bacterial Vaginosis through Enhanced Biofilm Removal and Avoidance of Resistance

**DOI:** 10.1128/aac.02319-21

**Published:** 2022-04-13

**Authors:** Christine Landlinger, Vera Oberbauer, Lenka Podpera Tisakova, Timo Schwebs, Rocío Berdaguer, Leen Van Simaey, Mario Vaneechoutte, Lorenzo Corsini

**Affiliations:** a BioNTech R&D (Austria) GmbH, Vienna, Austria; b University of Ghent, Laboratory Bacteriology Research, Department of Diagnostic Sciences, Faculty of Medicine & Health Sciences, Ghent, Belgium

**Keywords:** bacterial vaginosis, metronidazole resistance, *Gardnerella* biofilm, endolysin, alternative to antibiotic treatment

## Abstract

Antibiotics are the mainstay of therapy for bacterial vaginosis (BV). However, the rate of treatment failure in patients with recurrent BV is about 50%. Herein, we investigated potential mechanisms of therapy failure, including the propensity of resistance formation and biofilm activity of metronidazole (MDZ), clindamycin (CLI), and PM-477, a novel investigational candidate that is a genetically engineered endolysin with specificity for bacteria of the genus *Gardnerella*. Determination of the MIC indicated that 60% of a panel of 22 *Gardnerella* isolates of four different species were resistant to MDZ, while all strains were highly susceptible to CLI and to the endolysin PM-477. Six strains, all of which were initially susceptible to MDZ, were passaged with MDZ or its more potent hydroxy metabolite. All of them generated full resistance after 5 to 10 passages, resulting in MICs of >512 μg/mL. In contrast, only a mild increase in MIC was found for PM-477. There was also no cross-resistance formation, as MDZ-resistant *Gardnerella* strains remained highly susceptible to PM-477, both in suspension and in preformed biofilms. Strains that were resistant to MDZ in suspension were also tolerant to MDZ at >2,048 μg/mL when growing as biofilm. All strains were susceptible to PM-477 when grown as preformed biofilms, at minimum biofilm eradication concentrations (MBECs) in the range of 1 to 4 μg/mL. Surprisingly, the MBEC of CLI was >512 μg/mL for 7 out of 9 tested *Gardnerella* strains, all of which were susceptible to CLI when growing in suspension. The observed challenges of MDZ and CLI due to resistance formation and ineffectiveness on biofilm, respectively, could be one explanation for the frequent treatment failures in uncomplicated or recurrent BV. Therefore, the high efficacy of PM-477 in eliminating *Gardnerella* in *in vitro* biofilms, as well as its high resilience to resistance formation, makes PM-477 a promising potential alternative for the treatment of bacterial vaginosis, especially in patients with frequent recurrence.

## INTRODUCTION

Bacterial vaginosis (BV) is a very common disorder in women of reproductive age, with a prevalence estimated at 20 to 30% worldwide (1–3). It is caused by an imbalance in the microbiome of the vagina that results mostly in discharge, irritation, and odor. Although symptoms of a single episode of BV may be mild to moderate, those patients suffering from frequently recurring BV are prone to sequelae such as infertility and early spontaneous abortion (4–6), as well as an increased risk of preterm delivery and low birth weight (7, 8), and are at high risk of contracting sexually transmitted diseases, including HIV (9, 10). Besides these potentially severe physiological consequences, patients with recurring and strongly symptomatic BV report a strong impact on their sex life and overall quality of life and psychological well-being, including reduced self-esteem (11).

The current model of the etiology of BV focuses on the importance of *Gardnerella*. It postulates that *Gardnerella* bacteria form an adherent biofilm on the vaginal epithelium, in which other species can proliferate, resulting in a polymicrobial biofilm (12–16).

The recommended first-line therapy for BV is antibiotic treatment, predominately with metronidazole (MDZ) or clindamycin (CLI). However, treatment failure and recurrent disease are common problems (17, 18).

MDZ belongs to the group of nitroimidazoles and only gains its full activity when it is metabolized into its hydroxy metabolite (MDZ-OH). This limits the spectrum of activity to anaerobic (19) and microaerophilic (20) bacteria. MDZ is effective in quickly reducing BV symptoms, but is associated with a high recurrence rate of up to 60% within 6 months of treatment (18). In a clinical trial where patients with recurrent BV were treated with 0.75% MDZ vaginal gel over 16 weeks, the probability of lasting cure was 70% after 16 weeks (i.e., 30% of patients had symptoms at the end of the 16-week treatment period) and declined to 34% 12 weeks after the end of therapy (i.e., at week 28) (21). Besides a possible reinfection from sexual partners, the persistence of a residual infection has been postulated as a reason for recurrence, potentially due to the formation of a biofilm that protects BV-causing bacteria from antimicrobial therapy (12, 18, 22). Another reason may be antibiotic resistance of BV pathogens. In a clinical study, the treatment of bacterial vaginosis with CLI was associated with marked antimicrobial resistance among vaginal anaerobic bacteria, while resistance to MDZ was minimal (23, 24). Nonetheless, vaginal *Gardnerella* isolates showed high MDZ resistance rates (16, 25–27). Resistance to CLI was found in 36% of *Gardnerella* patient isolates by Castro et al. (16), but in none of the isolates in other studies (26, 27).

Endolysins are promising potential alternatives to the current antibiotics, due to their ability to remove biofilms, their low propensity to the development of resistance, and their specificity to individual genera or species of bacteria (28). As an example, an endolysin targeting Staphylococcus aureus showed only minimal decrease in susceptibility when passaged with sub-bacteriostatic concentrations for 26 days, whereas the same procedure using antibiotics increased antibiotic resistance drastically (29).

The genetically engineered endolysin PM-477 was recently described as a *Gardnerella*-specific, highly potent antimicrobial, which was shown to dissolve the *Gardnerella*-dominated biofilm on exfoliated human epithelial cells in vaginal swabs from patients with BV (26). In this study, we show that *Gardnerella* spp. quickly develop resistance to MDZ upon serial passaging for 8 to 9 rounds with sub-MICs. Our data also support the contention that MDZ and CLI are ineffective on the majority of tested *Gardnerella* isolates if they are grown as a biofilm. In contrast, PM-477 effectively inhibits bacterial growth also after 25 rounds of passaging, is fully active on MDZ-resistant *Gardnerella* strains, and is highly active on 48- to 72-h-old preformed biofilms. In summary, we provide further data on PM-477 as a preclinical candidate under investigation for BV, especially for women with recurrent BV who failed treatment with MDZ or CLI.

## RESULTS

### Fifty-nine percent of tested *Gardnerella* type strains and patient isolates are MDZ resistant.

The MICs of MDZ, tinidazole (TDZ), CLI, and PM-477 were determined for 22 *Gardnerella* and 5 *Lactobacillus* strains following the Clinical and Laboratory Standards Institute (CLSI) protocol for anaerobic bacteria (30). The resistance breakpoints defined by EUCAST for Gram-positive anaerobes are >4 μg/mL for both CLI and MDZ (31). However, topical intravaginal antibiotic administration is recommended for BV therapy, which allows the drugs to reach concentrations of active ingredients in the mg/mL range. Therefore, in this study, we used resistance breakpoints of ≥8 μg/mL and ≥32 μg/mL for CLI and MDZ, respectively, as previously described for BV-related bacteria (27). For *Gardnerella* treated with MDZ, we measured MICs in the range of 8 to >256 μg/mL, with a MIC value for 90% of strains (MIC_90_) above 256 μg/mL (Table 1; for further details, see Table S1 in the supplemental material). Thirteen out of 22 tested isolates (59%) had a MIC for MDZ at or higher than 32 μg/mL. With regard to the different *Gardnerella* species, MDZ resistance rates varied from 25% for Gardnerella vaginalis to 100% for Gardnerella leopoldii and Gardnerella swidsinskii–—however, given the small number of isolates tested per species, the species-specific resistance rates are unlikely to be representative. The MIC values for MDZ and TDZ were very similar, and largely the same isolates were resistant or susceptible (Table S1), indicating very similar mechanisms of activity and resistance formation. In contrast, all *Gardnerella* strains of all four species were highly susceptible to CLI (MIC_90_, 0.5 μg/mL; range, <0.06 to 2 μg/mL) as well as to the endolysin PM-477 (MIC_90_, 1 μg/mL; range, <0.03 to 1 μg/mL). Lactobacilli were not susceptible to PM-477, MDZ, or TDZ, while at least the Lactobacillus crispatus and Lactobacillus jensenii strains tested were susceptible to CLI. CLI had a largely bacteriostatic effect on the two Lactobacillus gasseri strains tested, as the MIC was 32 to 64 μg/mL, while the minimum bactericidal concentration at which 99.5% of isolates are killed (MBC_99.5_) was above 128 μg/mL (Table S1).

**TABLE 1:** MICs of metronidazole, clindamycin, and PM-477 for *Gardnerella* type strains and patient isolates^*a*^

Species (no. of isolates)	Antimicrobial agent	MIC (μg/mL)		No. (%) resistant
		Range	MIC_90_	
*G. vaginalis* (8)	MDZ	8 to 64	64	2 (25)
	CLI	<0.06 to 0.5	0.5	0
	PM-477	<0.03 to 0.5	0.5	ND

*G. leopoldii* (3)	MDZ	128 to >256	>256	3 (100)
	CLI	0.5	0.5	0
	PM-477	0.125 to 0.5	0.5	ND

*G. piotii* (7)	MDZ	8 to 128	128	4 (57)
	CLI	0.125 to 2	2	0
	PM-477	0.5 to 1	1	ND

*G. swidsinskii* (4)	MDZ	64 to >256	>256	4 (100)
	CLI	<0.06 to 0.5	0.5	0
	PM-477	0.03 to 0.25	0.25	ND

All *Gardnerella* (22)	MDZ	8 to >256	>256	13 (59)
	CLI	<0.06 to 2	0.5	0
	PM-477	<0.03 to 1	1	ND

a Resistance is defined as ≥32 μg/mL and ≥8 μg/mL for metronidazole (MDZ) and clindamycin (CLI), respectively. The MIC_90_ is defined as the MIC value for 90% of the tested strains. For PM-477, the resistance breakpoint is not defined; therefore, a percentage of isolates resistant is not defined (ND).

### Distinct killing of *Gardnerella* cells in biofilms by MDZ, CLI, and PM-477.

**FIG 1:**
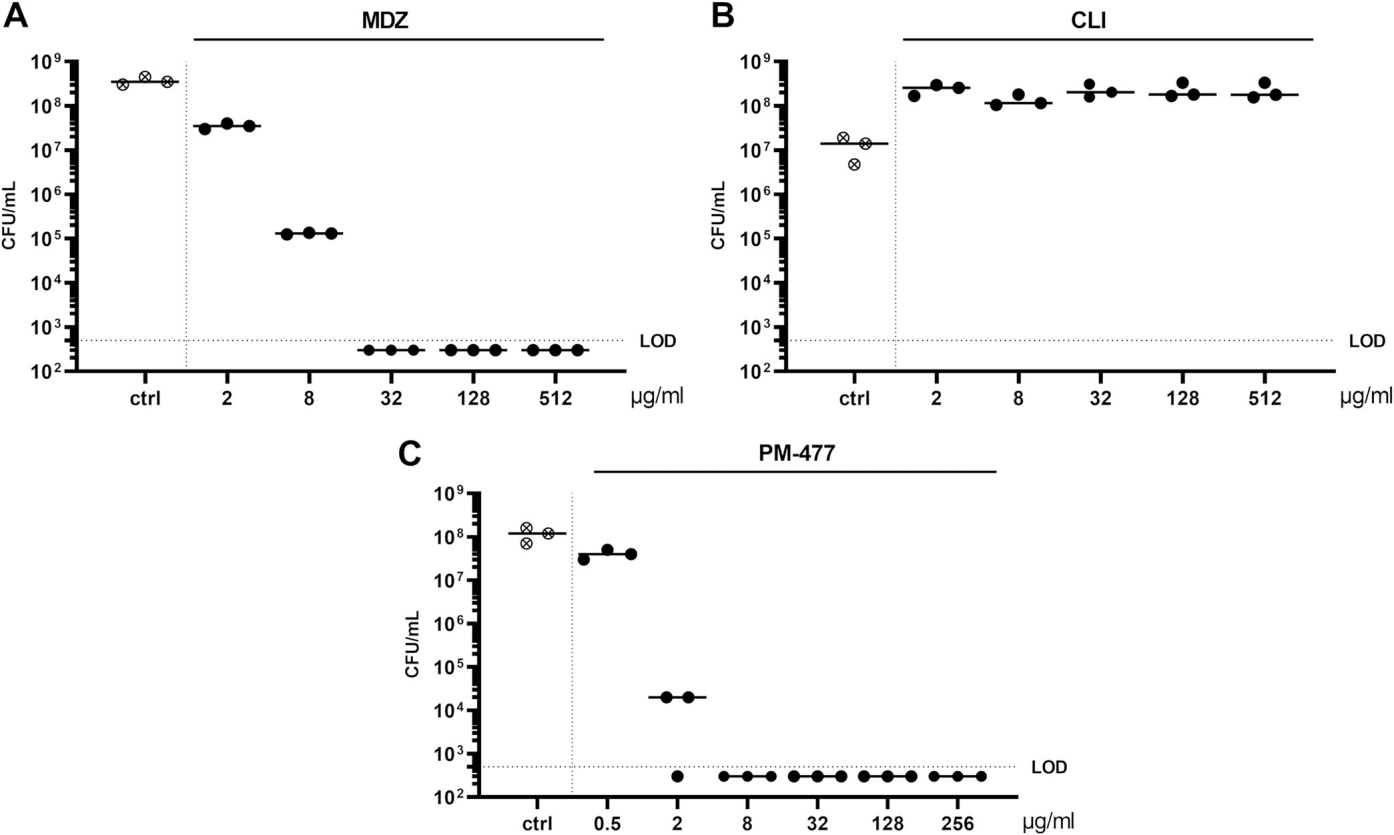
*G. vaginalis* (ATCC 14018^T^) grown in biofilm can be removed beyond the limit of detection (LOD) by metronidazole (MDZ) and PM-477, but not by clindamycin (CLI) treatment. Biofilms were grown for 40 h and then incubated for another 24 h with the indicated antimicrobial. For analysis, the antimicrobials were washed off, the biofilm was dislodged mechanically, and the surviving cells were enumerated in CFU/mL by quantitative plating. ctrl, control (medium only).

*Gardnerella*-dominated biofilms covering the human vaginal epithelial cells are now generally considered a hallmark of BV. Thus, we tested how effective antibiotics and PM-477 are in penetrating and killing *in vitro* preformed 40- to 72-h-old biofilms of various *Gardnerella* strains. Interestingly, *Gardnerella* strains, which are all highly susceptible to CLI in suspension (MICs ranging from 0.01 to1 μg/mL), become tolerant to CLI when grown as biofilms (Table 2 and Fig. 1), with a minimum biofilm eradication concentration (MBEC) of up to ≥512 μg/mL. MDZ removed the biofilms beyond the limit of detection (LOD) at MBECs ranging from 8 to 128 μg/mL, while the endolysin PM-477 was able to kill all six *Gardnerella* strains grown as biofilm at MBECs lower than any of the antibiotics (i.e., <2 to 32 μg/mL). The reduction in biofilm CFU of a representative *Gardnerella* strain (*G. vaginalis* ATCC 14018^T^) by MDZ, CLI, and PM-477 is depicted in Fig. 1. It should be noted that a reduction in CFU is a hint of but no proof for killing of the bacteria, as the treated cells could be slower growing or more difficult to dislodge. Another method frequently used to detect the reduction of biofilm biomass is crystal violet staining. This method was not used in this case, because killing of biofilm bacteria by antibiotics would not be expected to reduce the biomass of the biofilm. Instead, we chose a method by which all three antimicrobials can be compared.

**TABLE 2:** MBEC values of ancestor (nonpassaged) *Gardnerella* isolates

Ancestor *Gardnerella* isolate	MBEC (μg/mL)^*a*^		
	MDZ	PM-477	CLI
*G. vaginalis*			
ATCC 14018^T^	32	32	>512
UGent 09.07	32	16	>512
UGent BV50	128	2	>512
UGent BV111.5	32–128	8–32	>512
UGent FB049	2–8	0.5–2	>512

*G. swidsinskii*			
GS 9838-1^T^	32	<2	>512
UGent BV 7.1	32	32	<4

*G. piotii*			
UGent 18.01^T^	8	8	<4
UGent 21.28	8–32	2–8	>512

a MBEC, minimum biofilm eradication concentration; MDZ, metronidazole; CLI, clindamycin; ^T^, after strain designation indicates a type strain.

### *Gardnerella* spp. that are initially susceptible to MDZ quickly become resistant upon serial passaging.

**FIG 2:**
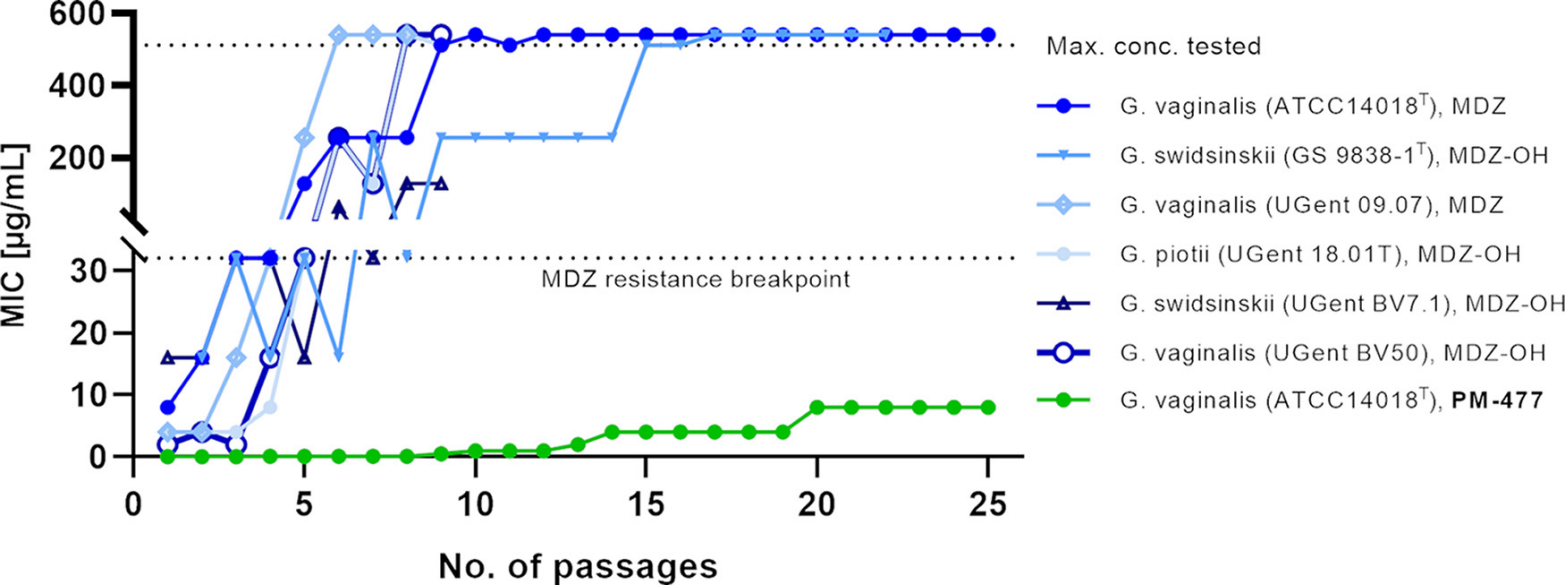
Resistance formation by serial passaging. Six *Gardnerella* strains were passaged for up to 25 days in the presence of metronidazole (MDZ) or the hydroxy metabolite of MDZ (MDZ-OH) at sub-MICs, as indicated in the figure key. One representative *Gardnerella* strain was passaged with PM-477 for 25 days, as indicated in the figure key. The MIC (μg/mL) was determined every day prior to the next round of passaging. ^T^, after strain designation indicates a type strain.

Six *Gardnerella* strains that were initially susceptible either to MDZ (*G. vaginalis* ATCC 14018^T^, MIC of 8 μg/mL) or at least to the more potent MDZ hydroxy metabolite (MDZ-OH) (MIC of between 2 and 16 μg/mL [data not shown]) were serially passaged in MDZ or MDZ-OH at sub-MICs, so that growth was impaired but not completely inhibited. Upon passaging, the MICs for both MDZ and the MDZ-OH metabolite increased strongly, and all *Gardnerella* strains reached the resistance breakpoint of ≥32 μg/mL within 5 rounds of passaging (Fig. 2, blue lines). After 9 rounds of passaging, 4 out of 6 strains could no longer be inhibited even by the maximal concentration in use (MIC of >512 μg/mL) (Fig. 2). In parallel, one representative of *G. vaginalis* (ATCC 14018^T^) was passaged 25 times with PM-477. The MIC of PM-477 increased only slightly over the 25 rounds of passaging, to 8 μg/mL (Fig. 2, green line).

### Passaged isolates with acquired MDZ resistance remain susceptible to PM-477.

**FIG 3:**
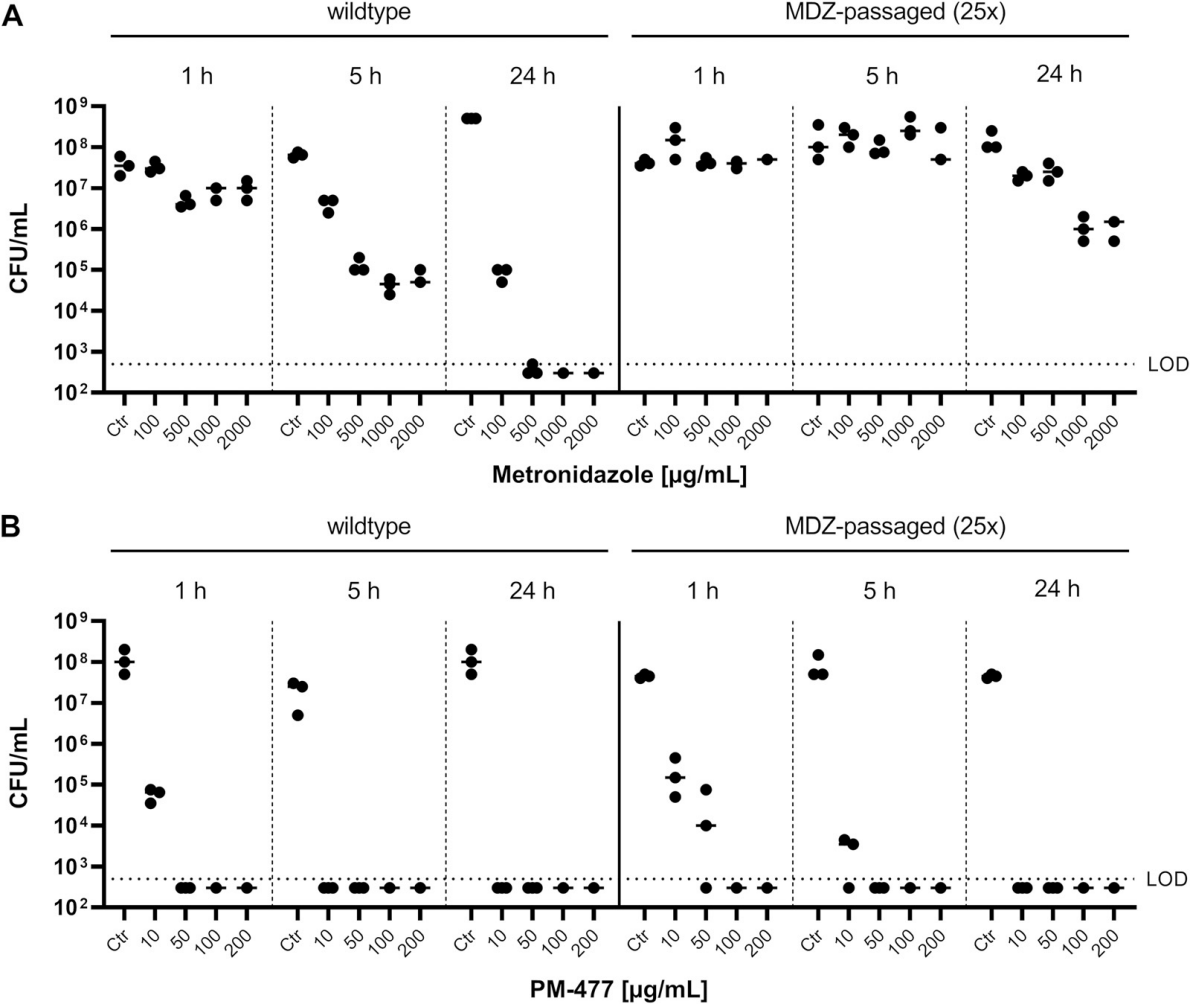
Lytic effect of metronidazole (MDZ) and PM-477 on wild-type and MDZ-passaged *G. vaginalis* (ATCC 14018^T^) cells in suspension. Wild-type cells naive to MDZ and the strain passaged with sub-MICs of MDZ for 25 rounds were treated with for 1, 5, and 24 h with MDZ (A) and PM-477 (B), respectively. Ctr, control (medium only); LOD, limit of detection.

Next, we tested to what extent the resistance of *Gardnerella* cells toward MDZ also impacts the susceptibility to the endolysin PM-477. *G. vaginalis* (ATCC 14018^T^) passaged for 25 days on MDZ was exposed to different concentrations of MDZ and PM-477 for 1, 5, and 24 h, and then the bactericidal effect was assessed by quantitative plating on chocolate (Choc) agar plates. For the ancestral strain, there is a clear dose and time dependency of viability upon exposure to MDZ (Fig. 3A). In contrast, the *G. vaginalis* strain passaged on MDZ can tolerate very high concentrations of MDZ—up to 2 mg/mL—for 1 and 5 h without any loss in viability, and the treatment with 2 mg/mL for 24 h resulted in only a 2-log reduction compared to the buffer-treated control. When *G. vaginalis* (ATCC 14018^T^) was exposed to PM-477, the susceptibilities were similar for the ancestral and the passaged strains (Fig. 3B). After 1 h of treatment, 10 μg/mL PM-477 reduced a suspension of 10^8^ CFU/mL by 3.0 and 2.8 log units for the ancestral and passaged strains, respectively. Also, the MIC values of PM-477 were very similar for ancestral and MDZ-passaged strains (Table 3). This indicates that the acquired resistance against MDZ does not interfere with the mode of action of the endolysin.

**TABLE 3:** Susceptibility of *Gardnerella* strains before and after passaging 8 to 9 times with sub-MICs of MZD or MDZ-OH^*a*^

*Gardnerella* strain	Passaging antibiotic	MIC (μg/mL)						MBEC (μg/mL) when passaged with:	
		Before passaging			After passaging				
		MDZ	MDZ-OH	PM-477	MDZ	MDZ-OH	PM-477	MDZ	PM-477
*G. vaginalis*									
ATCC 14018^T^	MDZ	8	2	0.06	>512	>512	0.13	>2,048	2–8
UGent 09.07^*b*^	MDZ-OH	64	8	0.25	>2,048	>512	0.25	>2,048	2–4
UGent BV50	MDZ-OH	32	4	0.5	>2,048	>512	0.25	>2,048	1–8

*G. swidsinskii*									
GS 9838-1^T^	MDZ-OH	256	16	0.03	>512	>512	0.06	>2,048	2
UGent BV 7.1	MDZ-OH	256	32	0.25	>2,048	>512	0.06	>2,048	2

*G. piotii* UGent 18.01^T^	MDZ-OH	32	8	1	>2,048	>512	2	>2,048	<1

a All MICs were determined after 48 h of incubation. MBEC, minimal biofilm radication concentration; MDZ, metronidazole; MDZ-OH, hydroxy metabolite of MDZ; ^T^, after strain designation indicates a type strain.

b *G. vaginalis* UGent 09.07 was passaged only 8 times with MDZ-OH.

**FIG 4:**
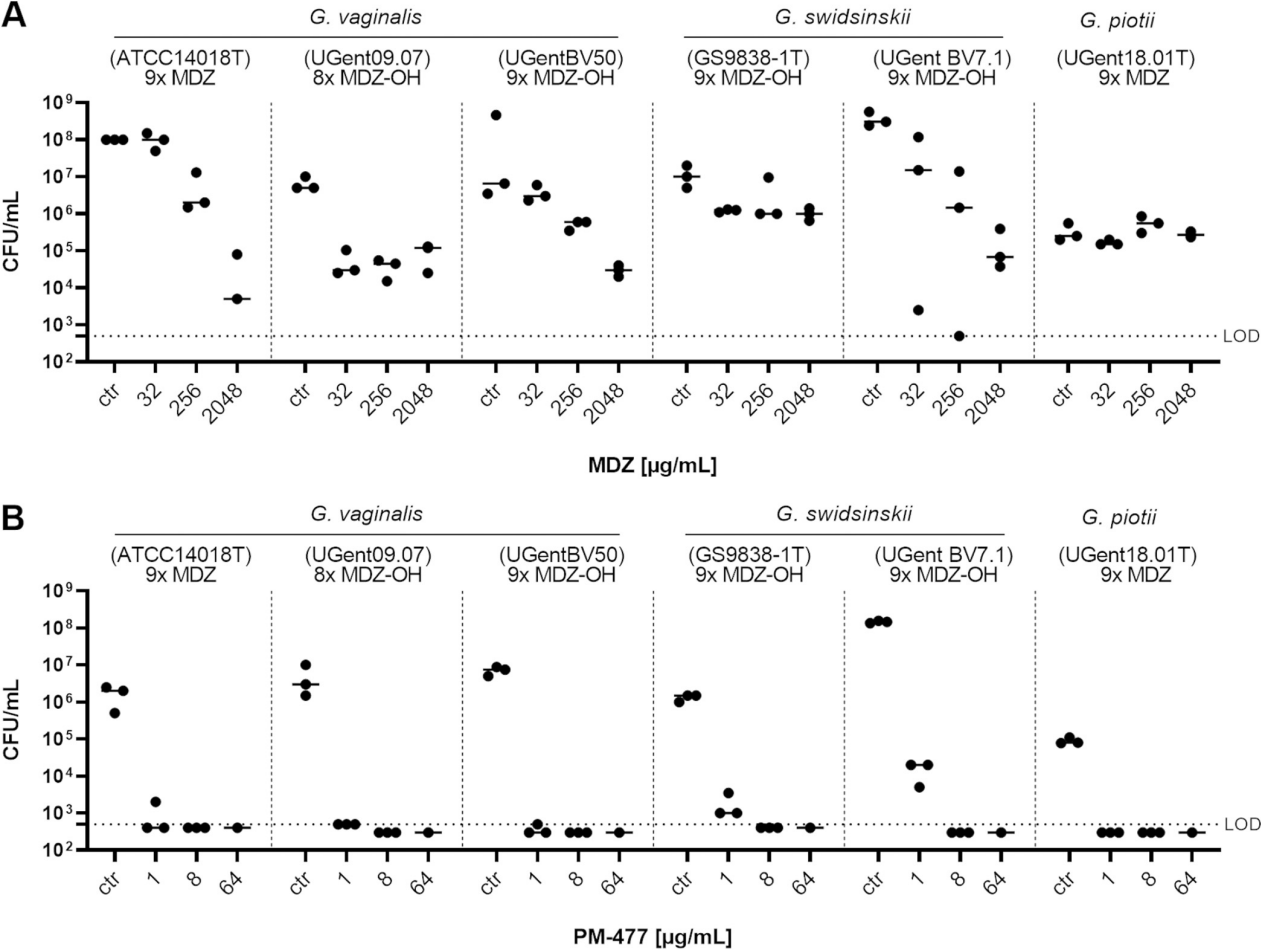
Strains passaged with metronidazole (MDZ) or its hydroxy metabolite (MDZ-OH), as indicated at the top of the panels, were grown as biofilm and treated with MDZ or PM-477. The 72-h preformed biofilms were treated for 24 h with different concentrations of MDZ (A) and PM-477 (B), as indicated on the *x* axis, and the surviving cells were quantified. ctr, control (medium only); LOD, limit of detection. The numbers at the top of each panel (e.g., 9× MDZ) indicate how many rounds the individual strain was passaged with sub-MICs of MDZ or MDZ-OH. ^T^, after strain designation indicates a type strain.

All MDZ- or MDZ-OH-treated passaged *Gardnerella* strains were still capable of forming biofilms similar in thickness and CFU count to their respective nonpassaged ancestors (data not shown). The MBEC of MDZ on *Gardnerella* strains passaged on MDZ or MDZ-OH was >2,048 μg/mL for all six passaged strains (Fig. 4A). Of these, only one *G. vaginalis* strain (ATCC 14018^T^) initially had a MIC below the resistance breakpoint. However, given that intravaginal MDZ (typically a 0.75% cream [i.e., 7.5 mg/mL]) can establish concentrations in the mg/mL range in vaginal fluid, we also passaged and tested strains with MICs above the EUCAST breakpoint (prepassaging MIC range, 8 to 256 μg/mL) (Table 3).

In contrast, all strains were highly susceptible to PM-477, with MBECs in the range of 1 to 8 μg/mL (Fig. 4B and Table 2).

## DISCUSSION

There is an unmet need for new BV therapies. First-line antibiotics used to treat BV have shown cure rates at 21 to 30 days of 40% to 60% for nonrecurring BV in clinical trials (32–34), and rates were consistently below 50% if only patients with a history of recurrence were included (18, 21).

In this study, we show that *Gardnerella* isolates with MICs initially below 32 μg/mL for MDZ or for the more potent hydroxy metabolite MDZ-OH built up resistance to MDZ or MDZ-OH above 32 μg/mL within 5 rounds of passaging and became tolerant to concentrations of MDZ up to 2 mg/mL upon only 5 to 15 rounds. *Gardnerella* biofilms of all MDZ-susceptible strains could be removed beyond the LOD with MDZ treatment, with MBEC values of 8 to 128 μg/mL. While after passaging with MDZ, these strains could still grow as biofilms, these biofilms were no longer susceptible to MDZ, and the isolates had MBEC values of >2 mg/mL. It can be hypothesized that the fast resistance formation against MDZ observed here *in vitro* might also occur in BV patients during a course of treatment. In fact, it was reported that failure of MDZ therapy is associated with a history of BV, including its treatment (18, 35). Therefore, each further course of MDZ treatment might increase the tolerance of the bacteria for this antibiotic. Because we found that the resistance statuses across strains were comparable for MDZ and TDZ, these findings might hold for all nitroimidazoles, including secnidazole.

MDZ has been associated with fast resistance formation before: e.g., for Helicobacter pylori (36), Clostridioides difficile (37), and *Prevotella* (38). The relevance of MDZ resistance formation for the therapy failure of BV has been a matter of debate. In two studies published in 2004 and 2005, MDZ-resistant vaginal anaerobic bacteria, such as *Gardnerella* or *Prevotella*, were not found after intravaginal MDZ therapy (23, 24). However, these studies also showed that *Gardnerella* was not eradicated in more than 60% of patients. In more recent studies, MDZ-resistant *Gardnerella* isolates were found consistently (16, 25–27).

We also show that the synthetic endolysin PM-477 is not prone to resistance development, even after 25 passages (Fig. 2). This property may be inherent for endolysins in general (29), which has been explained by the fact that endolysins with an enzymatically active domain of the GH25 muramidase family, as is the case for PM-477, cleave conserved bonds in the peptidoglycan cell wall, which bacteria cannot easily change (39). In contrast, many mechanisms are known to lead to resistance to MDZ (40–47). Importantly, in our study, *Gardnerella* strains passaged on MDZ did not increase tolerance to PM-477, indicating that the mechanisms of action of PM-477 and resistance formation against MDZ are very different.

Bacteria of the genus *Gardnerella* form an adherent biofilm on the vaginal epithelium in which other species can proliferate, resulting in a polymicrobial biofilm (12–14, 16, 48). Therefore, we also evaluated the potency of PM-477, MDZ, and CLI on various *Gardnerella* strains grown as *in vitro* biofilms. Surprisingly, we found that when grown as biofilm, 7/9 strains tested were tolerant to 24 h of treatment with a concentration of CLI of >512 μg/mL, while the MIC and MBC_99.5_ values of the same strains in suspension were <1 μg/mL. In fact, a strongly reduced activity of CLI on *Gardnerella* growing as biofilm *in vitro* was shown previously (49). CLI is a bacterial protein synthesis inhibitor with a mainly bacteriostatic effect (50), so that the reduced killing efficacy of CLI on bacteria growing as biofilm may be explained by their low metabolic and protein synthesis activities (compared to planktonic bacteria in growth phase, for which MICs are usually measured). Although the natural polymicrobial biofilm found in BV patients will certainly differ in many aspects from the monospecies biofilms studied here *in vitro*, our findings provide a potential explanation for therapy failure upon CLI treatment, as it is likely that the metabolic activity of *Gardnerella* is critical for its susceptibility to CLI. Furthermore, CLI would negatively affect the population of lactobacilli, thus further destabilizing the vaginal microbiome. In fact, the clinical cure rate of CLI after 25 to 39 days reported in clinical trials never exceeded 39% (33), and there are no reports of its efficacy on patients with recurrent BV.

While our data provide potential explanations for the observed (22) failure of antibiotics to remove the biofilm associated with BV, sexual reinfection can also be a reason for recurrence (51). The treatment of episodes that recur due to sexual reinfection might, however, also fail, given the issues with MDZ and CLI described here, emphasizing the need for more effective alternatives.

In summary, this study highlights potential explanations for the failure of BV therapy with both first-line antibiotics: CLI is inactive on most *Gardnerella* strains when they grow as biofilm but affects lactobacilli, while MDZ (and potentially all other nitroimidazoles) allows fast resistance formation on all *Gardnerella* strains tested. In contrast, the genetically engineered investigational candidate lysin PM-477 kills *Gardnerella* cells in preformed *in vitro* biofilm, does not affect beneficial lactobacilli, and does not allow bacteria to become tolerant. With its high selectivity, its bactericidal potency on biofilms, and its robustness against resistance formation, we conclude that PM-477 is a potential candidate for the treatment of patients suffering from BV, particularly recurring BV.

## MATERIALS AND METHODS

### Bacterial isolates and culture conditions.

*Gardnerella* isolates of different species (52) were obtained from the Laboratory of Bacteriology, University of Ghent, Belgium. These isolates include strains purchased from culture collections and fresh isolates from BV patients obtained from the University Clinic Bruges. *Gardnerella* isolates were grown on chocolate (Choc) agar plates (Becton, Dickinson) under anaerobic conditions in an anaerobic chamber equipped with anaerobic atmosphere generation bags (Sigma-Aldrich) for 48 h. All isolates were cultured in New York City broth III (NYCB), consisting of 10 mM HEPES (Sigma-Aldrich), 15 g/L proteose peptone (Sigma-Aldrich), 3.8 g/L yeast extract (Thermo Fisher Scientific), 86 mM sodium chloride (Carl Roth), and 28 mM α-d-glucose (Sigma-Aldrich), supplemented with 10% horse serum (HS) (Thermo Fisher Scientific). Table S1 lists all *Gardnerella* and *Lactobacillus* isolates studied.

### Preparation of PM-477.

The endolysin PM-477 was engineered and produced as previously described (26). Briefly, PM-477 was recombinantly expressed in Escherichia coli BL21(DE3). Protein purification was performed by affinity chromatography on a nickel-nitrilotriacetic acid (Ni-NTA) HisTrap column. The protein was eluted with a mixture of 50 mM MES (morpholineethanesulfonic acid) (Carl Roth) at pH 7, 150 mM NaCl (Carl Roth), and 150 to 500 mM imidazole fractions (2-fold dilutions). Compared to the preparation used in the 2021 study by Landlinger et al. (26), the N-terminal His tag was cleaved off by digestion with 1:100 (wt/wt) 3C protease. The removed tag and the protease were separated from PM-477 by anion-exchange chromatography. The untagged protein was concentrated (if needed) and dialyzed against MES buffer (50 mM MES [pH 5.50], 200 mM NaCl, 8 mM MgSO_4_) (Sigma-Aldrich).

The protein concentration was determined at the optical density at 260/280 nm (OD_260/280_) or by using the Pierce bicinchoninic acid (BCA) protein assay kit (Thermo Fisher Scientific). Purified PM-477 aliquots of 1,000 μL and at a concentration of 0.7 mg/mL were stored at −80°C until the moment of use.

### Culture-based assessment of bactericidal activity.

Bacterial suspensions (OD_600_ of 0.1, corresponding to approximately 10^7^ to 10^8^ CFU/mL) were prepared by scraping the cells from cultures confluently grown on agar plates and diluting them into NYCB plus 10% HS (pH 5.5). Reactions were performed in triplicate by mixing 10 μL of endolysin (200 μg/mL) with 90 μL bacterial suspension in the wells of a 96-well plate. Ten microliters of MES buffer without the endolysin was used as a control. The 96-well reaction plate was incubated anaerobically at 37°C for 5 h. Ten-fold dilution series (10^−1^ to 10^−6^) of the cell reaction mixtures were prepared in NYCB plus 10% HS, and 2 μL of each dilution was spotted onto Choc agar plates. After anaerobic incubation at 37°C for 48 h, colonies were counted, CFU/mL were calculated, and the log_10_ reduction compared to the MES buffer-treated control was determined.

### MIC assessment.

The MIC, which is a standard measure of the activity of antimicrobials, was determined according to the 2018 Clinical and Laboratory Standards Institute protocol *Methods for Antimicrobial Susceptibility Testing of Anaerobic Bacteria* (30). Bacterial suspensions of 10^5^ to 10^6^ CFU/mL in NYCB plus 10% HS were treated with a 2-fold dilution series of either PM-477 or the antibiotic clindamycin (clindamycin hydrochloride) (Sigma-Aldrich), both tested at a starting concentration of 64 μg/mL, or the antibiotic metronidazole (MDZ) (Gatt-Koller) and its hydroxy metabolite 1(2-hydroxyethyl)-2-hydroxy-methyl-5-nitroimidazole (MDZ-OH) (Sigma-Aldrich), starting at concentrations of 2,048 or 512 μg/mL, respectively, or tinidazole (TDZ) (Sigma-Aldrich), starting at 128 μg/mL. Controls for growth in the absence of antimicrobials were also included. OD_600_ was recorded by a microplate reader (Tecan, Grödig, Austria) after incubation at 37°C for 48 h or, for some fast-growing strains, after incubation for 24 h. Absence of growth was defined as an OD_600_ of ≤0.14.

### Serial passaging for resistance formation profiling.

Bacterial suspensions of 10^5^ to 10^6^ CFU/mL in NYCB plus 10% HS were prepared for the treatment with a 2-fold dilution series of either the endolysin PM-477 produced as described previously (26), PM-477 with modifications defined in this article, metronidazole (MDZ), or 1(2-hydroxyethyl)-2-hydroxy-methyl-5-nitroimidazole (MDZ-OH) (Sigma-Aldrich). The final volume of the reaction was 100 μL, and the reactions were performed in triplicate in 384-well plates. The OD_600_ was measured using a microplate reader (Tecan, Grödig, Austria) to generate time point 0-h baseline values. The reaction mixtures were incubated anaerobically for 24 h, and then the OD_600_ was measured again, and the MIC was measured as the lowest concentration with an OD_600_ of <0.14. The bacterial cell suspension treated with the highest concentration of antimicrobial for which growth was detected (sub-MIC) was diluted to 10^5^ to 10^6^ CFU/mL and treated anew with a dilution series of antimicrobials. This process was repeated for a maximum of 25 rounds. In each round, the input CFU/mL was determined by quantitative plating.

### Biofilm formation and MBEC determination.

*Gardnerella* (see the individual figures for strain specification) was resuspended in brain heart infusion broth supplemented with 2% (wt/vol) gelatin, 0.5% yeast extract (wt/vol), 0.1% starch (wt/vol) and 0.25% glucose (wt/vol) (sBHIG). Passaged strains were resuspended in unbuffered NYCB supplemented with 1% glucose (wt/vol) (sNYCB). The OD_600_ was set to 0.1 (approximately 10^7^ to 10^8^ CFU/mL), and the cell suspension was diluted 1:10 in growth medium as input for biofilm formation. A total of 200 μL of the respective bacterial suspensions was added to 96-well flat-bottom plates (tissue-culture treated; Sigma-Aldrich). The biofilms were grown under anaerobic conditions at 37°C for 40 to 72 h, depending on the isolate. Subsequently, the supernatant was removed, and the biofilm was treated with antimicrobials dissolved in sBHIG (at pH 5 for treatment with PM-477 and unbuffered for treatment with antibiotics) in 100 μL and incubated anaerobically at 37°C for another 24 h. After the treatment, the biofilms were washed twice with 200 μL 1× phosphate-buffered saline (PBS) and dissolved by vigorous pipetting (40 times up and down). Serial dilutions of the dissolved cells were spotted onto chocolate agar plates. The plates were incubated anaerobically for 2 to 3 days, and the minimum biofilm eradication concentrations (MBEC) of the endolysin or the antibiotics in use were calculated.

### Statistical analysis.

Where appropriate, data were log normalized prior to applying statistical tests (e.g., for CFU/mL values and as indicated in the figure legends). When only two groups were compared, the unpaired two-tailed *t* test was used, as indicated in the respective figure legends. Multiple groups were compared by two-tailed one-way analysis of variance (ANOVA). The software used for statistical analyses was GraphPad Prism8. Differences between groups were considered statistically significant when *P* was <0.05.
