# Supplementary material for: Preclinical Data on the Gardnerella-Specific Endolysin PM-477 Indicate Its Potential to Improve the Treatment of Bacterial Vaginosis through Enhanced Biofilm Removal and Avoidance of Resistance
Source: Antimicrob Agents Chemother. 2022 Apr 13;66(5):e02319-21. doi: 10.1128/aac.02319-21 (PMC9112913; doi:10.1128/aac.02319-21)
Supplement: Supplemental file 1 — Table S1. Download aac.02319-21-s0001.pdf, PDF file, 0.2 MB [file aac.02319-21-s0001.pdf]

Supplementary Table 1: Susceptibility of *Gardnerella* strains to the antimicrobials PM-477, metronidazole (MDZ), clindamycin (CLI) and tinidazole (TDZ). Minimum inhibitory concentration (MIC) and minimum bactericidal concentration 99.5 ($MBC_{99.5}$ is defined here as the minimum concentration that reduces CFU by 99.5% within 24 h of treatment) values are reported for each strain. Abbreviations: n.d., not determined; SJHB, St. Jan Hospital Bruges, Belgium; UG, University Gent, Belgium.

***Gardnerella* strain/ isolate**				**Antimicrobials [µg/ml]**							
				PM-477		MDZ		CLI		TDZ	
Name	Origin	Strain	Clade	MIC	$MBC_{99.5}$	MIC	$MBC_{99.5}$	MIC	$MBC_{99.5}$	MIC	$MBC_{99.5}$
G. vaginalis (Gv9)	ATCC	ATCC 14018(T)	IB	0.06	0.25	8	16	0.25	0.5	128 (R)	>128 (R)
G. vaginalis (Gv1)	UG	UGent 09.07	IA	0.25	1	64 (R)	512 (R)	0.25	0.25	>128 (R)	>128 (R)
G. vaginalis (Gv5)	UG	UGent 09.01	IB	<0.03	0.125	8	512* (R)	0.13	0.25	4	8
G. vaginalis (Gv8)	UG	UGent 25.49	IB	0.03	0.125	8	32 (R)	<0.06	0.25	4	32 (R)
G. vaginalis (BV50)	SJHB	UGent BV50.1	n.d.	0.5	1	32 (R)	128 (R)	0.25	0.5	64 (R)	>128 (R)
G. vaginalis (BV111)	SJHB	UGent BV111.5	n.d.	0.25	0.5	8	64 (R)	0.125	2	4	128 (R)
G. vaginalis (FB049)	UG	UGent FB049-01	n.d.	0.25	0.25	16	64 (R)	0.5	1	8	64 (R)
G. vaginalis (FB061)	UG	UGent FB061-03	n.d.	0.5	2	8	32 (R)	0.25	1	4	32 (R)
G. leopoldii (Gl11)	UG	UGent 09.48	II	0.125	0.5	128 (R)	>128 (R)	0.5	1	128 (R)	>128 (R)
G. leopoldii (BV217)	SJHB	UGent BV217.1	n.d.	0.25	1	256 (R)	512 (R)	0.5	1	n.d.	n.d.
G. leopoldii (BV13)	SJHB	UGent BV13.2	n.d.	0.5	2	>128 (R)	>128 (R)	0.5	1	>128 (R)	128 (R)
G. piotii (Gp17)	UG	UGent 18.01(T)	III	1	4	32 (R)	64 (R)	0.5	1	64 (R)	>128 (R)
G. piotii (Gp22)	UG	UGent 21.28	III	1	2	64 (R)	>128 (R)	0.25	0.5	>128 (R)	>128 (R)
G. piotii (P80275)	SJHB	UGent P80275	III	0.5	1	16	64 (R)	0.5	1	32 (R)	128 (R)
G. piotii (FB041)	SJHB	UGent FB041	III	0.5	1	32 (R)	64 (R)	1	2	64 (R)	128 (R)
G. piotii (BV049)	SJHB	UGent BV049.1	n.d.	1	1	8	32 (R)	0.125	0.125	n.d.	n.d.
G. piotii (BV140)	SJHB	UGent BV140.2	n.d.	1	2	128 (R)	>512 (R)	2	64 (R)	n.d.	n.d.
G. piotii (BV154)	SJHB	UGent BV154.1	n.d.	0.5	1	8	32 (R)	0.5	0.5	n.d.	n.d.
G. swidsinskii (Gs23)	UG	GS 10234	IV	0.063	0.125	64 (R)	>128 (R)	0.25	0.25	>128 (R)	>128 (R)
G. swidsinskii (Gs24)	UG	GS 9838-1(T)	IV	0.03	0.06	256 (R)	>512 (R)	<0.06	0.5	>128 (R)	>128 (R)
G. swidsinskii (BV139)	SJHB	UGent BV139.3	n.d.	0.125	0.125	256 (R)	256 (R)	0.125	0.25	n.d.	n.d.
G. swidsinskii (BV7)	SJHB	UGent BV7.1	n.d.	0.25	0.25	256 (R)	>512 (R)	0.5	1	128 (R)	>128 (R)

Lactobacillus spp.				**Antimicrobials [µg/ml]**							
				PM-477		MDZ		CLI		TDZ	
Name	Origin	Strain	Clade	MIC	$MBC_{99.5}$	MIC	$MBC_{99.5}$	MIC	$MBC_{99.5}$	MIC	$MBC_{99.5}$
Lactobacillus crispatus	DSM	DSM 20584		>256	>256	>128	>128	4	4	>128	>128
Lactobacillus gasseri	DSM	DSM 20077		>256	>256	>128	>128	64	>128	>128	>128
Lactobacillus gasseri	DSM	DSM 20243		>256	>256	>128	>128	32	>128	>128	>128
Lactobacillus jensenii	UG	PB2003-073-T2-3		>256	>256	>128	>128	0.25	4	>128	>128
Lactobacillus jensenii	UG	PB2003-013-T2-2		>256	>256	>128	>128	0.25	4	>128	>128
